# Supplementary material for: FREQ‐NESS Reveals the Dynamic Reconfiguration of Frequency‐Resolved Brain Networks During Auditory Stimulation
Source: Adv Sci (Weinh). 2025 Apr 10;12(20):2413195. doi: 10.1002/advs.202413195 (PMC12120751; doi:10.1002/advs.202413195)
Supplement: Supplementary file 1 — Supporting Information [file ADVS-12-2413195-s001.pdf]

ADVANCED SCIENCE Open Access

Supporting Information

for *Adv. Sci.*, DOI 10.1002/advs.202413195

FREQ-NESS Reveals the Dynamic Reconfiguration of Frequency-Resolved Brain Networks During Auditory Stimulation

Mattia Rosso, Gemma Fernández-Rubio, Peter Erik Keller, Elvira Brattico, Peter Vuust, Morten L Kringelbach and Leonardo Bonetti**

Supplementary Information for

FREQ-NESS Reveals the Dynamic Reconfiguration of Frequency-Resolved Brain Networks During Auditory Stimulation

Rosso, M.[1,2], Fernández-Rubio, G.[1], Keller, P.[1], Brattico, E.[1,3], Vuust, P.[1], Kringelbach, M. L.[1,4,5], Bonetti, L.[1,4,5*]*

[1] *Center for Music in the Brain, Department of Clinical Medicine, Aarhus University & The Royal Academy of Music, Aarhus/Aalborg, Aarhus, Denmark*

[2] *IPEM Institute for Systematic Musicology, Ghent University, Ghent, Belgium*

[3] *Department of Education, Psychology, Communication, University of Bari Aldo Moro, Italy*

[4] *Centre for Eudaimonia and Human Flourishing, Linacre College, University of Oxford, Oxford, United Kingdom*

[5] *Department of Psychiatry, University of Oxford, Oxford, United Kingdom*

*Corresponding authors: mattia.rosso@clin.au.dk and leonardo.bonetti@psych.ox.ac.uk

Co-authors contacts:
gemmafr@clin.au.dk
p.keller@clin.au.dk
elvira.brattico@clin.au.dk
vuust@clin.au.dk
morten.kringelbach@psych.ox.ac.uk

ORCID:
0000-0003-2349-1386 (Mattia Rosso)

Supplementary analyses

To evaluate the robustness and generalizability of the main results, we conducted a series of supplementary analyses following the same structure as the main study. These analyses aimed to validate the findings across different datasets, pre-processing pipelines, and methodological variations, ensuring the reliability and soundness of the FREQ-NESS analytical pipeline under varying conditions. Specifically, we computed the following additional analyses:

1. Replication of frequency-resolved brain networks in two new datasets with Resting State (RS)
2. Replication of frequency-resolved brain networks in shorter fragments (30, 60, 120, 180, and 240 seconds of recording) of the original datasets for both RS and Passive Listening (PL).
3. Replication of frequency-resolved brain networks for both RS and PL of the original dataset computed on the source reconstructed data using gradiometers only.
4. Comparison between PCA and GED within the FREQ-NESS pipeline.

In all additional analyses and replications, FREQ-NESS was applied to characterize the temporal and spatial configurations of brain networks operating at specific frequencies and to examine their consistency across datasets. As in the original analysis, we used GED to extract eigenvectors and eigenvalues from voxel-level data matrices reconstructed via beamforming. The primary output of FREQ-NESS remains consistent with the main analysis, comprising a landscape of frequency-specific networks characterized by the distribution of eigenvalues and spatial activation patterns. The analysis was performed across the entire frequency spectrum (0.2 to 97.6 Hz) in 1.2 Hz intervals. For reasons of readability, parsimony and direct comparison with the main results, the Supplementary Figures show the network landscape of the top three components from 0.2 Hz to 40.9 Hz. Moreover, we provide all information related to the statistical analyses in **Table S4**.

Whenever both RS and PL were included in the analysis, signed-rank Wilcoxon tests were performed to test for significant differences in the network landscapes across experimental conditions. Replicating the main analysis, a two-tailed test of the eigenvalues was performed testing PL against RS, for each component and each frequency. For this test, we assumed that significant changes could be found in both directions, due to the possible emergence or suppression of frequency-specific brain networks resulting from the auditory stimulation. False-discovery rate (FDR) correction was applied to all statistical tests to define a conservative significance threshold adjusted for multiple comparisons.

These supplementary analyses extend and reinforce the insights obtained from the main results, providing additional evidence for the consistency and reproducibility of the observed network dynamics across independent samples and variations in the processing parameters.

1. Replication on independent datasets (RS condition)

The present replication was designed to validate the RS network landscape using two independent datasets. Specifically, we aimed to enhance replicability by selecting datasets considerably larger than the original sample. Notably, *Supplementary Dataset 1* was chosen to address replicability in a sample with a narrow age range, as opposed to the relatively broad range in our original dataset. This RS dataset was gathered as part of a broader project that led to multiple studies (e.g., [1]) and has not been published previously. *Supplementary Dataset 2* was specifically chosen to further evaluate replicability, even with minor variations in data pre-processing. A large part of this RS dataset was used for different analyses, which are reported in [2].

Supplementary Dataset 1 comprised 5 minutes of resting-state data recorded with eyes open from 37 participants (N = 37; 18 females, 19 males) with an age range of 18 to 25 years (mean = 21.89; SD = 2.05). All pre-processing steps were identical to those used in the main analyses reported in the manuscript.

Supplementary Dataset 2 also comprised 5 minutes of resting-state data recorded with eyes open from 68 participants (N = 68; 34 females, 34 males) with an age range of 18 to 42 years (mean = 24.95; SD = 4.24). The MEG pre-processing pipeline was carried out with slight modifications to test the robustness of the method under varying parameters. The MEG pre-processing pipeline followed the steps carried out in our original dataset. In addition, the pre-processing for this dataset also involved high-pass filtering of the data with a 0.1 Hz cut-off, and down-sampling to 150 Hz. These two steps were performed immediately after MaxFilter, following the detailed pipeline reported in [2].

The results showed that, for both replication datasets, the distribution of explained variance across the frequency spectrum replicated the one observed in the RS condition of the original dataset. Specifically, the network landscape revealed a 1/f exponential decay of explained variance in the low delta range and local maxima in the alpha (10.9 Hz) and beta (22.9 Hz) frequency ranges. The spatial activation patterns showed the typical topographies expected from the brain at rest, namely the broad mesial distribution associated with the DMN for low frequencies not engaged in stimulus processing (2.4 Hz and 4.8 Hz), parieto-occipital distribution for the alpha network (10.9 Hz), and sensorimotor involvement for the beta network (22.9 Hz). Notably, while frequencies on the left side of the alpha peaked exhibit parieto-occipital activation (8.4 Hz and 9.6 Hz), the higher end of the range was characterized by sensorimotor activation (12.1 Hz). In the beta range, the explained variance peaked at 22.9 Hz, and the spatial pattern showed activation limited to sensorimotor regions.

These findings confirm the robustness of FREQ-NESS in characterizing brain network dynamics in resting-state conditions alone. The networks landscapes from the RS of both datasets are illustrated in **Figure S2**.

2. **Replication on shorter fragments of the dataset (RS and PL conditions)**

In order to evaluate the robustness of our main analysis to the amount of data provided as input, we conducted an additional replication analysis on shorter segments of the original dataset, corresponding to 30, 60, 120, 180, and 240 seconds (for reference, the original dataset consisted of 300 seconds of recordings). All segments started at the same initial time as the entire original dataset. The aim of this supplementary analysis was to determine the minimum recording duration required to reliably estimate a frequency-specific network landscape and detect significant differences across RS and PL conditions. To this end, statistical comparisons across conditions were performed as described above.

For *30 seconds*, significant changes in the first eigenvalue alone were found for the following frequencies: 2.4 Hz (1st component: $p < 0.001$, FDR-corrected) and 4.8 Hz (1st component: $p < 0.001$, FDR-corrected).

For *60 seconds*, significant changes in the first eigenvalue alone were found for the following frequencies: 2.4 Hz (1st component: $p < 0.001$, FDR-corrected) and 4.8 Hz (1st component: $p < 0.001$, FDR-corrected).

For *120 seconds*, significant changes in the first three eigenvalues were found for the following frequencies: 2.4 Hz (1st component: $p < 0.001$, FDR-corrected), 4.8 Hz (1st component: $p < 0.001$, FDR-corrected; 2nd component: $p < 0.001$, FDR-corrected), 7.2 Hz (1st component: $p < 0.001$, FDR-corrected), 9.6 Hz (2nd component: $p < 0.001$, FDR-corrected; 3rd component: $p < 0.001$, FDR-corrected), and 10.9 Hz (3rd component: $p < 0.001$, FDR-corrected).

For *180 seconds*, significant changes in the first three eigenvalues were found for the following frequencies: 2.4 Hz (1st component: $p < 0.001$, FDR-corrected), 4.8 Hz (1st component: $p < 0.001$, FDR-corrected; 2nd component: $p < 0.001$, FDR-corrected), 9.6 Hz (2nd component: $p < 0.001$, FDR-corrected; 3rd component: $p < 0.001$, FDR-corrected), 10.9 Hz (3rd component: $p < 0.001$, FDR-corrected), and 20.49 Hz (1st component: $p < 0.001$, FDR-corrected).

For *240 seconds,* significant changes in the first three eigenvalues were found for the following frequencies: 2.4 Hz (1st component: $p < 0.001$, FDR-corrected; 2nd component: $p < 0.001$, FDR-corrected), 4.8 Hz (1st component: $p < 0.001$, FDR-corrected; 2nd component: $p < 0.001$, FDR-corrected), and 9.6 Hz (1st component: $p < 0.001$, FDR-corrected; 3rd component: $p < 0.001$, FDR-corrected).

In summary, as shown in **Figure S3**, the results demonstrated that the network landscape could be reliably replicated with recordings as short as 30 seconds. The distribution of explained variance across the frequency spectrum and the associated spatial activation patterns remained consistent with those observed in the full-length recordings for both RS and PL conditions. However, only the

emergence of the primary auditory network at the stimulation frequency (2.4 Hz) and its harmonic (4.8 Hz) survived the FDR correction for durations of 30 and 60 seconds. The suppression of the alpha networks survived the FDR correction for durations of 120 seconds and above. With a duration of 240 seconds, we achieved full convergence with the main results.

Notably, the key features of the RS condition, such as the 1/f exponential decay in the delta range and local maxima in the alpha (10.9 Hz) and beta (22.9 Hz) ranges, were preserved even at shorter durations. Similarly, the PL condition retained its characteristic network landscape features, including prominent auditory network activation at the stimulation frequency and its harmonics. One notable artifactual effect encountered at the shortest durations is the steepening of the 1/f component, arguably due to the direct relationship between signal duration and frequency resolution. Since the Rayleigh frequency equals 1/T, where T is the signal duration expressed in seconds, the estimation of the lowest frequencies gets more reliable as the signal duration increases [3].

In summary, while the most prominent effects such as the emergence of auditory networks attuned to the stimulation survive a strict correction for multiple comparisons, we highlight that slightly longer recordings are preferred to detect more nuanced changes in the network landscape such as the shift in the alpha peak. As a general guideline, we recommend a minimum of 120 seconds (where differences in alpha reach statistical significance), and a recommended duration of 240 seconds to obtain a full convergence of the results with our original findings.

These findings provide additional methodological insights, further corroborating the feasibility and consistency of FREQ-NESS, and offering practical guidelines for incorporating the pipeline into larger experimental protocols.

3. Replication on gradiometers (RS and PL conditions)

To validate the robustness of the results with respect to the choice of MEG sensors type (i.e. magnetometers or gradiometers) to reconstruct the anatomical sources, the entire FREQ-NESS analysis pipeline was replicated on the original dataset using only gradiometers for source reconstruction (instead of magnetometers in the original analysis shown in the main manuscript). This replication aimed to assess the consistency of the network landscapes obtained from gradiometers with those derived from magnetometers in our main results. One participant was removed from the analysis due to technical issues during the recording, which affected only the gradiometers.

The network landscapes obtained using gradiometers exhibited the same features as those observed with magnetometers. In the RS condition, the distribution of explained variance revealed the characteristic 1/f exponential decay in the delta range and local maxima in the alpha (10.9 Hz) and beta (22.9 Hz) frequency ranges. Spatial activation patterns mirrored those derived from

magnetometers, including the broad mesial distribution associated with the DMN in the delta range (2.4 Hz and 4.8 Hz), parieto-occipital activation in the alpha range (10.9 Hz), and sensorimotor activation in the beta range (22.9 Hz).

In the PL condition, the network landscapes retained their key features, including the emergence of auditory networks at the stimulation frequency (2.4 Hz) and its harmonic (4.8 Hz), the shift of the alpha peak (12.1 Hz) and related activation in sensorimotor areas, and sensorimotor activation in the beta range (22.9 Hz). Spatial patterns confirmed prominent auditory cortex activation, as well as secondary auditory regions consistent with the findings derived from magnetometers.

When statistically comparing RS and PL, significant changes in the first two eigenvalues were found for the following frequencies: 2.4 Hz (1st component: $p < 0.001$, FDR-corrected; 2nd component: $p < 0.001$, FDR-corrected), 4.8 Hz (1st component: $p < 0.001$, FDR-corrected; 2nd component: $p < 0.001$, FDR-corrected), and 9.6 Hz (1st component: $p = 0.0038$; 2nd component: $p < 0.001$, FDR-corrected). All results replicated the statistical findings reported in the main analysis, with the minor exception that only the second component remained significant after FDR correction at 9.6 Hz.

These results indicate that the methodology is robust to the choice of MEG sensor type, returning consistent findings. Visual representations of the network landscapes obtained with gradiometers are provided in **Figure S4** for both RS and PL conditions.

4. Additional analysis: FREQ-NESS based on PCA

To explore a simpler alternative to GED, we implemented a version of FREQ-NESS based on principal component analysis (PCA) as an additional analysis pipeline. In this alternative approach, the multivariate brain voxel data were narrowband filtered across all the frequencies considered in the original analysis (0.2 Hz to 97.5 Hz). The same Gaussian filters used in the main analysis were employed for each frequency. Subsequently, PCA was applied independently to the filtered data across all frequencies to extract frequency-specific brain networks. Unlike GED, where the eigendecomposition is based on contrasting narrowband and broadband covariance matrices, PCA was applied exclusively to the narrowband covariance matrices.

In short, PCA was carried out as follows. After narrowband filtering, the data was centered by subtracting the mean of each brain voxel timeseries from the narrowband dataset X, as represented below:

$$\bar{X} = X - \mu$$

where μ is the mean vector of X.

Then, the covariance matrix C was computed on the centered data, as shown below:

$$C = \frac{1}{n-1} \bar{X}^T \bar{X}$$

where n is the number of data points.

Then, the eigenvalue equation for the covariance matrix was solved to find eigenvalues and eigenvectors:

$$CW = W\Lambda$$

where W is the eigenvector matrix (set of weights for each principal component) and Λ is the diagonal matrix of the corresponding eigenvalues (which we normalized in percent units to indicate the amount of variance explained by each component).

Finally, we computed the spatial projection of the components (spatial activation patterns A) in brain voxel space. This was done by multiplying the weights (the eigenvectors w in this case) by the covariance matrix C, as shown below:

$$A = w^T C$$

Figure S5 presents the network landscape generated by this alternative approach for both RS and PL conditions. Two key qualitative differences emerged when implementing FREQ-NESS with PCA compared to the original GED approach. First, there was a pronounced 1/f offset, which masked the emergence of frequency-specific peaks in the network landscape. Second, the components were sorted in a mixed manner based on explained variance. This likely occurred because PCA, despite using narrowband filtered data as input, prioritizes variance maximization over frequency specificity, leading to suboptimal separation of periodic components. For the same reason, we observed that the spatial activation pattern characteristic of the DMN persistently mixed with the topographies at each frequency. This further reinforces the notion that narrowband filtering alone does not ensure de-mixing of frequency-specific brain networks.

Statistical comparisons between RS and PL conditions revealed relatively small p-values; however, they did not survive FDR correction for multiple comparisons. The results are reported as follows: 2.4 Hz (1st component: $p = 0.0008$; 2nd component: $p = 0.0188$), 4.8 Hz (1st component: p

= 0.0435; 2nd component: p = 0.04386), and 9.6 Hz (1st component: p = 0.0059; 2nd component: p = 0.0912). While these results exhibit trends consistent with the original GED-based analysis, they also underscore the limitations of PCA in achieving frequency specificity due to its reliance on a variance-maximization criterion.

In summary, while PCA offers a simpler solution for linear decomposition, it proved suboptimal in systematically separating networks based on frequency specificity. This highlights the superiority of GED, which is explicitly designed to maximize this criterion. Future studies should systematically compare GED with a larger set of multivariate approaches [4] to further evaluate their relative strengths and potential applications in neuroscience.

Supplementary Tables

The supplementary tables are available at the following link:

https://doi.org/10.5281/zenodo.14917255

Table S1. Complete statistical results – variance explained

The table reports the results of the signed-rank Wilcoxon tests (n = 26) contrasting the variance explained by the first two generalized eigendecomposition (GED) components for passive listening (PL) versus resting state (RS). Z-values and p-values are reported, along with the threshold for the p-values computed by the False Discovery Rate (FDR) correction for multiple comparisons.

Table S2. Complete statistical results – cross-frequency coupling

The table reports the results of the signed-rank Wilcoxon tests (n = 26) contrasting the cross-frequency coupling between the phase of the first two generalized eigendecomposition (GED) components across the amplitude of the first component of all frequencies (starting at 14.9 Hz). This was done independently for both passive listening (PL) and resting state (RS). Z-values and p-values are reported, along with the threshold for the p-values computed by the False Discovery Rate (FDR) correction for multiple comparisons.

Table S3. Top eigenvalues – cumulative variance explained

This table presents the cumulative variance explained by the top 10 eigenvalues for each frequency in the resting state (RS) and passive listening (PL) conditions. The cumulative variance is reported incrementally: first for the leading eigenvalue alone, then for the sum of the first and second eigenvalues, then for the first three eigenvalues combined, and so forth, up to the top 10 eigenvalues. This approach quantifies the proportion of variance captured by the most prominent frequency-specific network components, thereby facilitating comparisons between conditions and across the frequency spectrum.

Table S4. Complete statistical results for all additional analyses – variance explained

The table reports the statistical results performed for each of the additional analyses described in the *Supplementary Information* section and illustrated in **Figures S3$_{a\text{-}e}$**, **S4**, and **S6**. Consistent with the main analysis, statistical testing relied on signed-rank Wilcoxon tests (n = 26) contrasting the variance explained by the first three generalized eigendecomposition (GED) components for passive listening (PL) versus resting state (RS). Z-values and p-values are reported, along with the threshold for the p-values computed by the False Discovery Rate (FDR) correction for multiple comparisons.

Supplementary Figures

The supplementary figures, along with the main figures, are provided in high definition at the following link:

https://doi.org/10.5281/zenodo.14917255

a

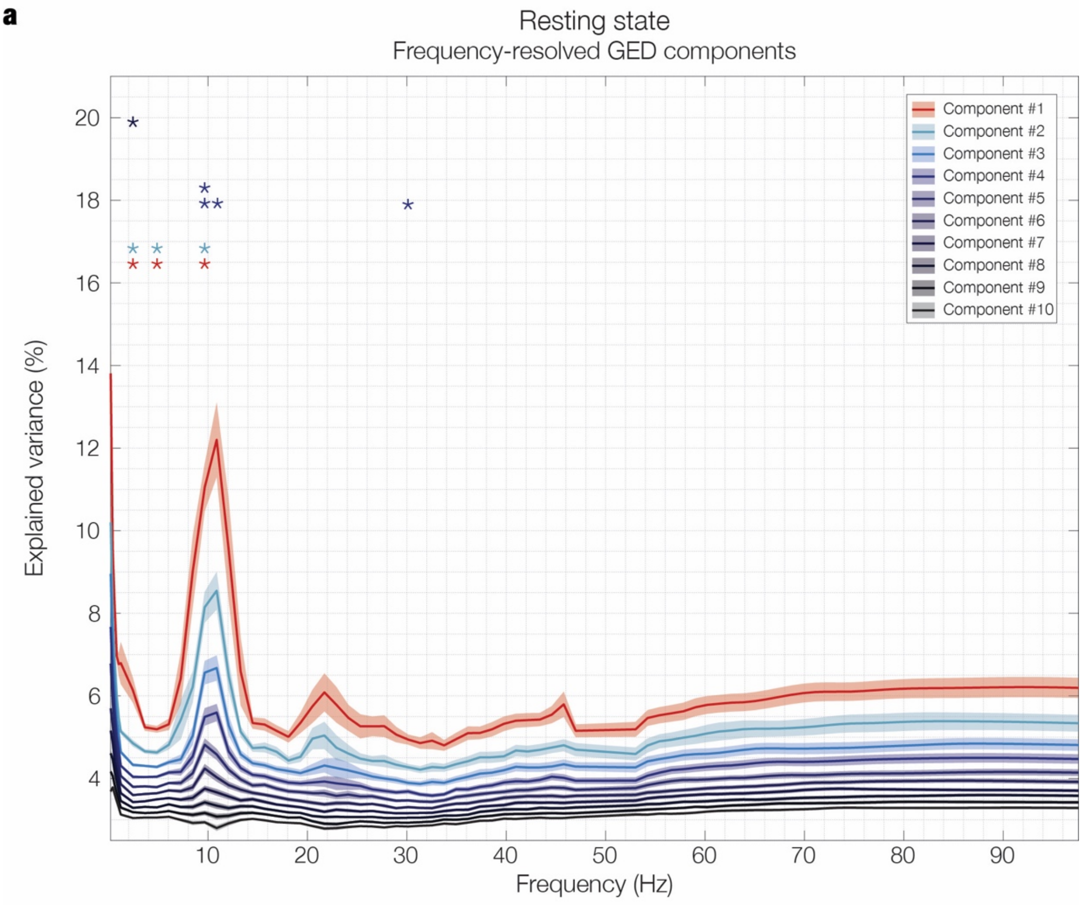

Resting state
Frequency-resolved GED components
Explained variance (%)
Frequency (Hz)
Component #1
Component #2
Component #3
Component #4
Component #5
Component #6
Component #7
Component #8
Component #9
Component #10

b

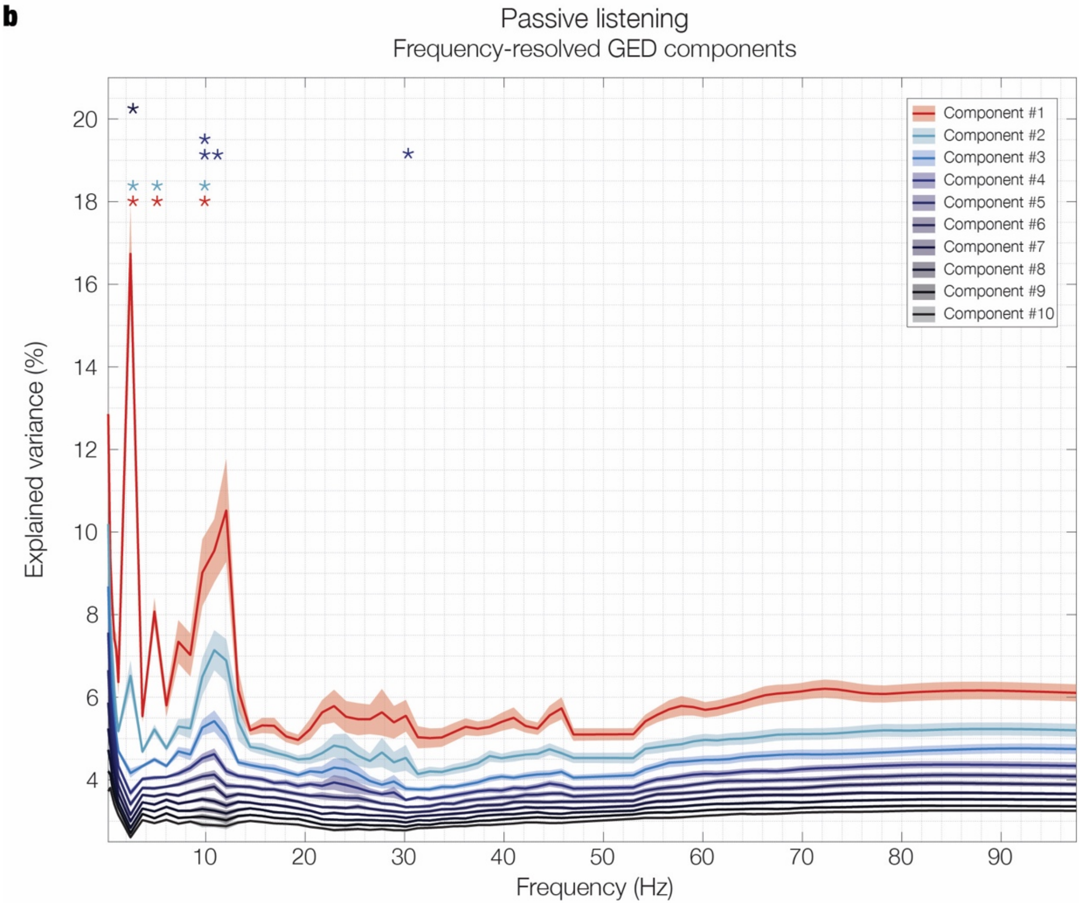

Passive listening
Frequency-resolved GED components
Explained variance (%)
Frequency (Hz)
Component #1
Component #2
Component #3
Component #4
Component #5
Component #6
Component #7
Component #8
Component #9
Component #10

Figure S1: Network landscape: eigenspectrum during rest and passive listening (full spectrum).

This figure illustrates the frequency-resolved ***eigenspectrum*** *estimated via FREQ-NESS in two experimental conditions: (a) resting state and (b) passive listening. The eigenspectrum shows the eigenvalues, which represent the percentage of variance explained by the associated GED components, plotted across frequencies. The solid lines indicate the mean normalized eigenvalues across participants, while the shaded areas represent the standard error of the mean (SEM). This is the same as* ***Figure 2****, but it displays the full frequency spectrum, emphasizing that no significant peak in variance explained is observed in the large gamma range. Frequencies ranging from 48 to 52 Hz were interpolated to prevent distortions of the landscape caused by power line interference at 50 Hz.*

a

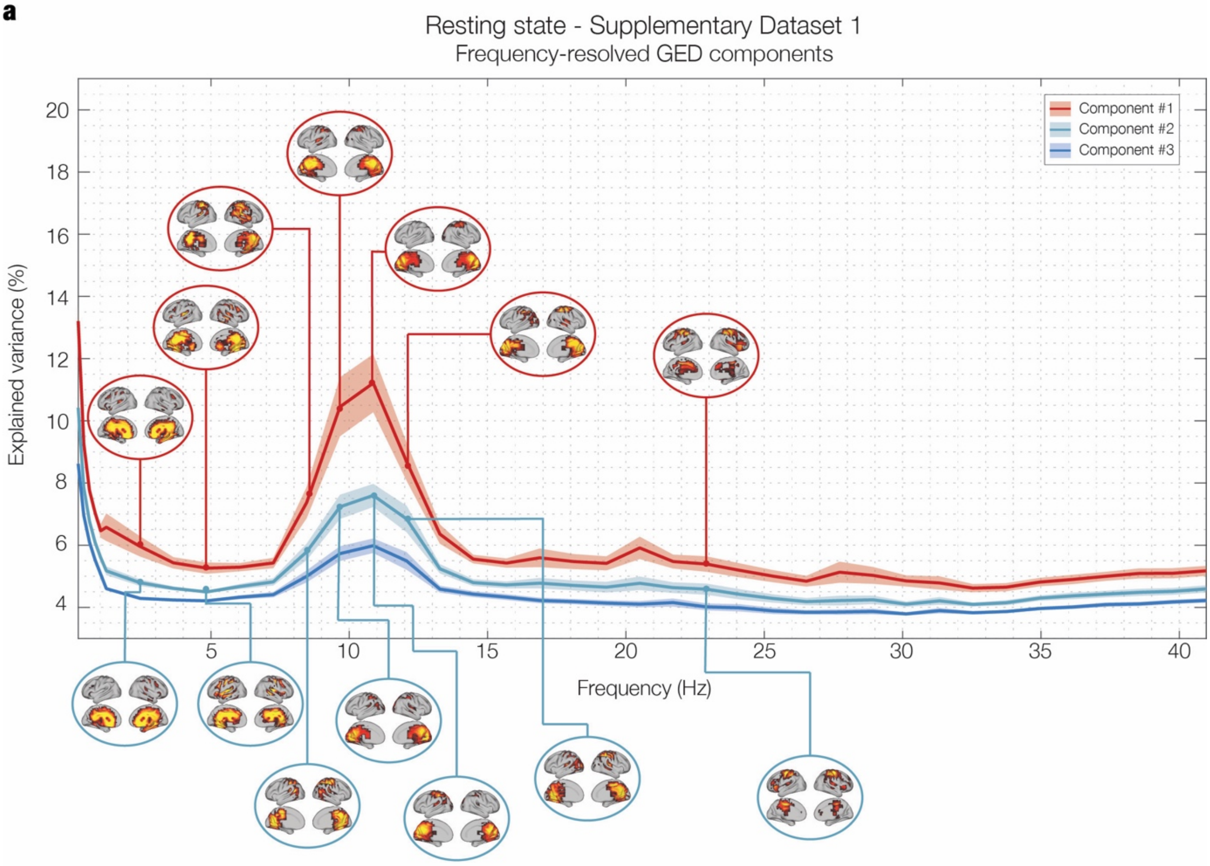

Resting state - Supplementary Dataset 1
Frequency-resolved GED components
Component #1
Component #2
Component #3
Explained variance (%)
Frequency (Hz)

b

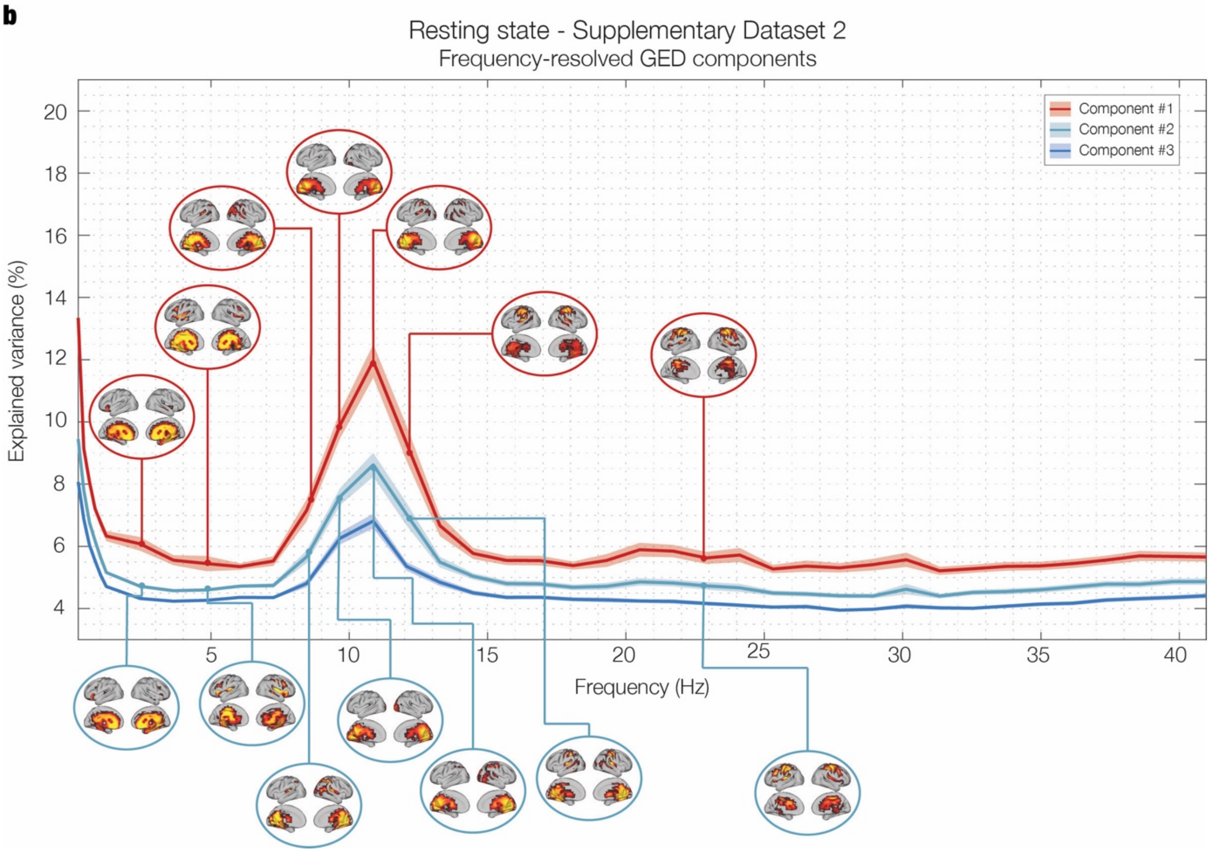

Resting state - Supplementary Dataset 2
Frequency-resolved GED components
Component #1
Component #2
Component #3
Explained variance (%)
Frequency (Hz)

Figure S2. Network landscape: Replication on independent supplementary datasets (RS condition).

This figure illustrates the frequency-resolved brain networks estimated via FREQ-NESS for a 5-minute RS condition, in two independent datasets: (a) Supplementary Dataset 1 and (b) Supplementary Dataset 2. The purpose of these datasets is to provide a replication of the resting-state network landscape in different and larger samples. The first element of the network landscape is the ***eigenspectrum****, which represents the eigenvalues as percentages of variance explained by the corresponding GED components, plotted across the frequency range. Solid lines denote the mean normalized eigenvalues across participants, with shaded regions indicating the standard error of the mean (SEM). The second element showcases the* ***spatial activation patterns*** *linked to the top GED components at frequencies of particular relevance for our study. These patterns illustrate the contribution of each brain voxel to the respective network. For clarity and consistently with the main results (see* ***Figure 2*** *in the main text), the eigenspectra are displayed for the first three GED components (ranked by explained variance) across a segment of the frequency spectrum (0.2 to 40.9 Hz), while the spatial topographies of the first two components are provided for frequencies of interest.* ***a – Supplementary Dataset 1****: The network landscape reveals a 1/f exponential decay of explained variance in the low delta range and local maxima in the alpha (10.9 Hz) and beta (22.9 Hz) frequency ranges. The spatial activation patterns show the typical topographies expected from the brain at rest, namely the broad mesial distribution associated with the DMN in the delta range (2.4 Hz and 4.8 Hz are shown, for consistency with the original dataset), parieto-occipital distribution for the alpha network (10.9 Hz), and sensorimotor involvement for the beta network (22.9 Hz). Notably, while frequencies on the left side of the alpha peak exhibit parieto-occipital activation (8.4 Hz and 9.6 Hz), the higher end of the range is characterized by sensorimotor activation (12.1 Hz). In the beta range, the explained variance peaks at 22.9 Hz, and the spatial pattern shows activation limited to sensorimotor regions.* ***b – Supplementary Dataset 2****: The network landscape exhibits the same features described above. A subtle difference is observed in the eigenvalue distribution around the alpha peak at 9.6 Hz, which appears sharper compared to the main dataset and Supplementary Dataset 1. This difference is likely due to the significantly larger sample size of this dataset (68 participants compared to 26 and 37 participants, respectively), resulting in a more precise separation of the most prominent center frequency in the low alpha range. In conclusion, these network landscapes provide a robust replication of the FREQ-NESS pipeline in resting-state, supporting its consistency in estimating a landscape of frequency-specific brain networks at rest.*

a

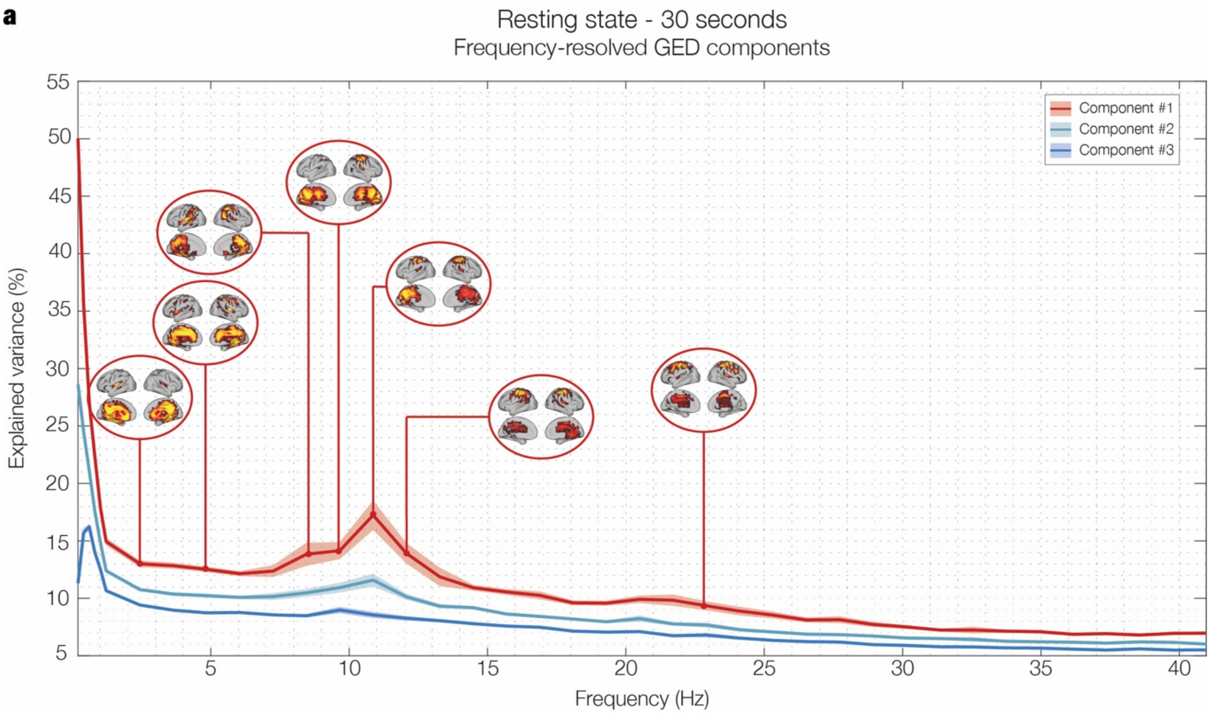

Resting state - 30 seconds
Frequency-resolved GED components
Explained variance (%)
Frequency (Hz)
Component #1
Component #2
Component #3

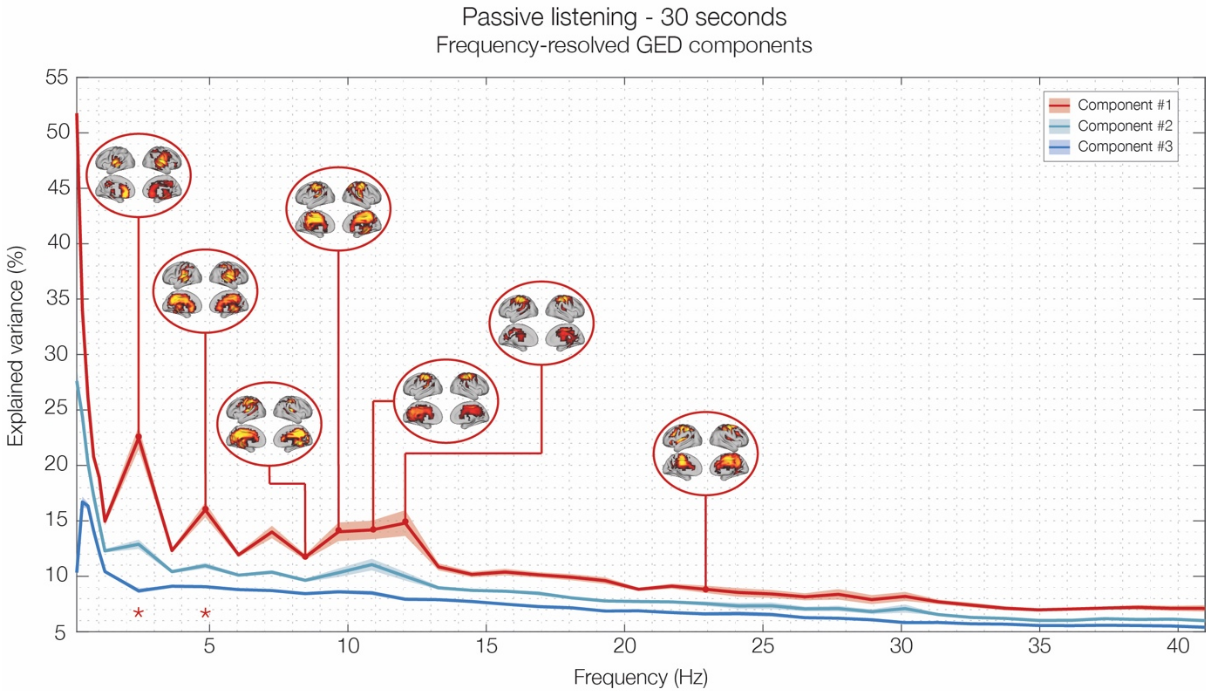

Passive listening - 30 seconds
Frequency-resolved GED components
Explained variance (%)
Frequency (Hz)
Component #1
Component #2
Component #3

b

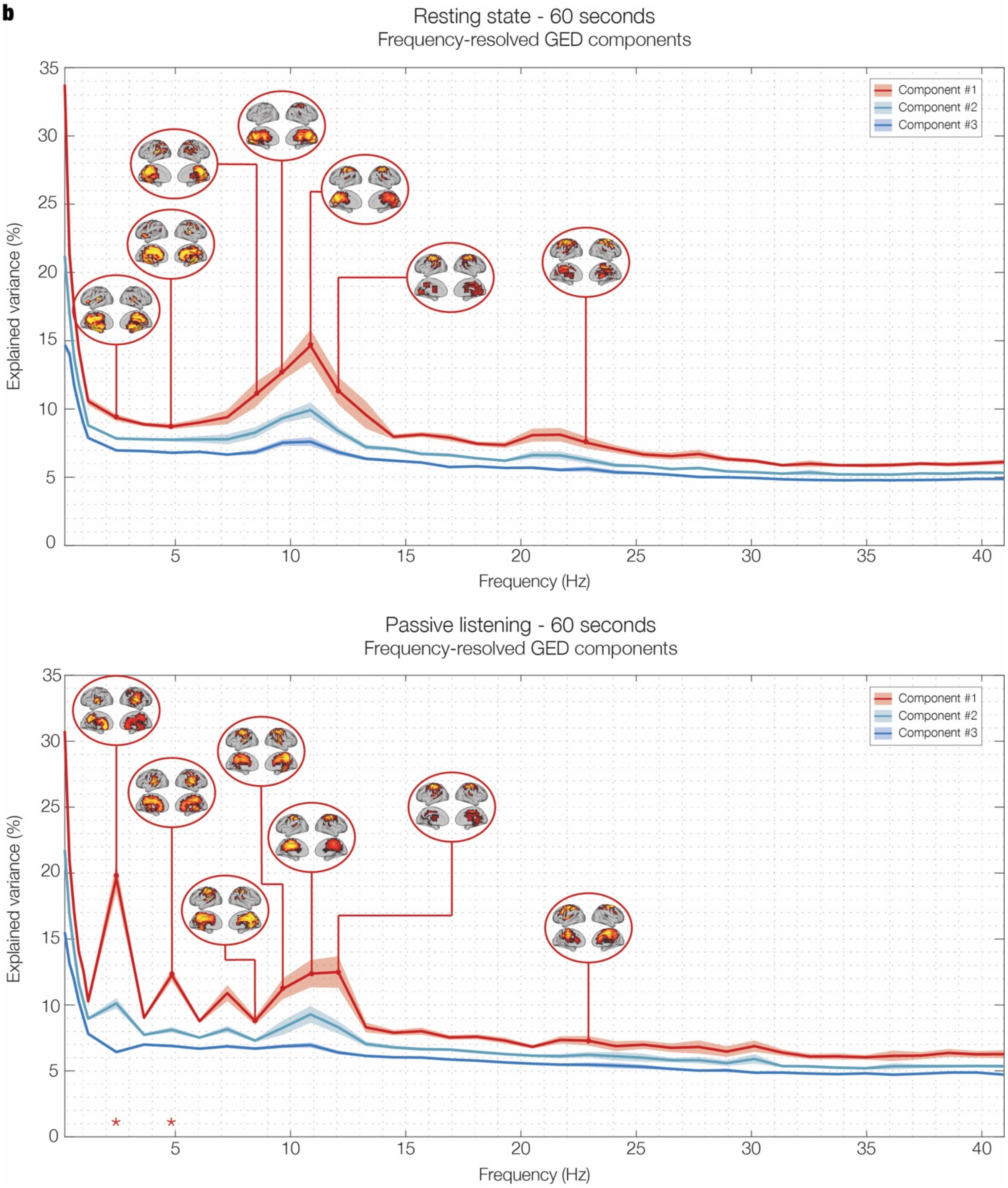
Resting state - 60 seconds
Frequency-resolved GED components
Explained variance (%)
Frequency (Hz)
Component #1
Component #2
Component #3
Passive listening - 60 seconds
Frequency-resolved GED components
Explained variance (%)
Frequency (Hz)
Component #1
Component #2
Component #3

c

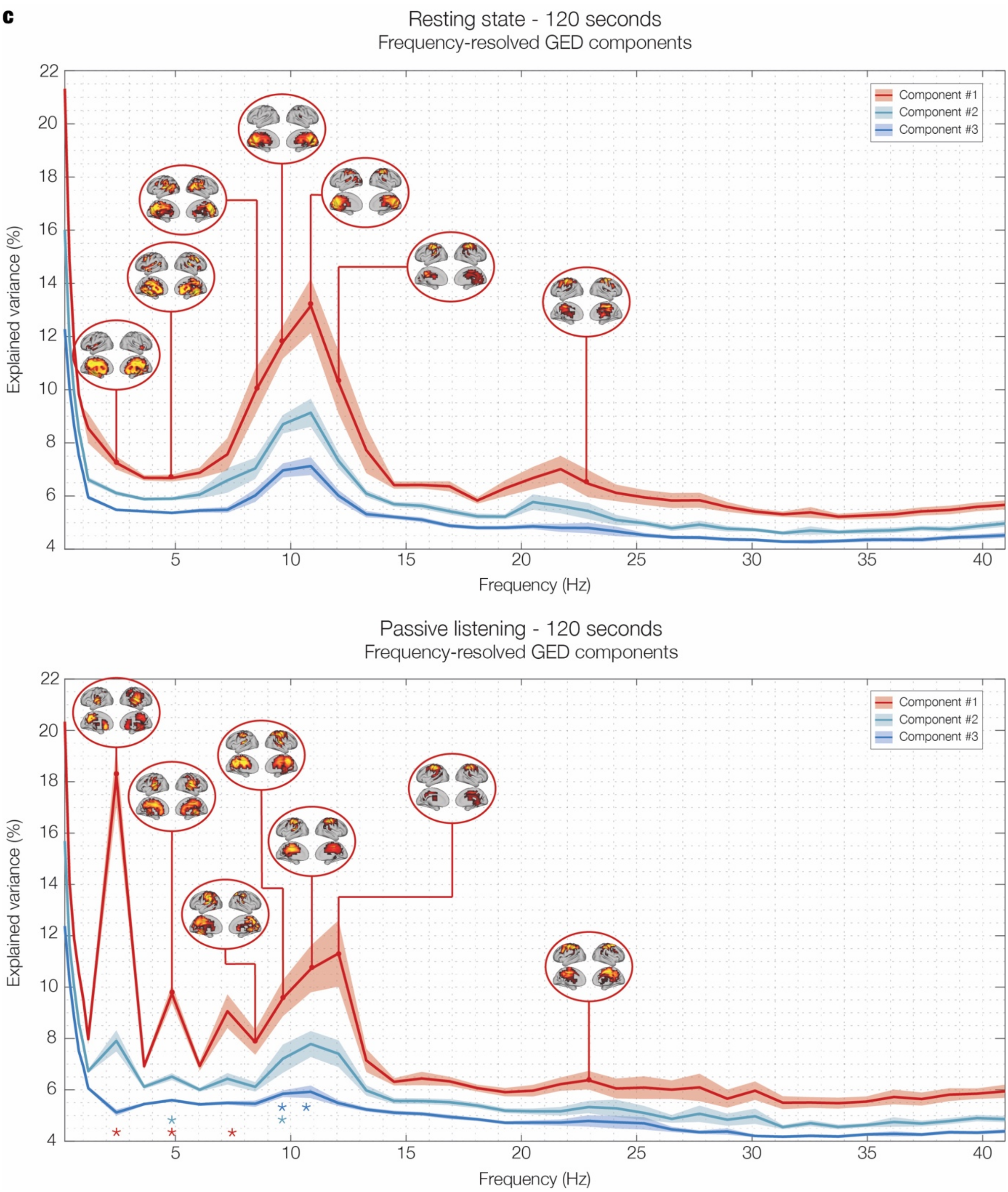
Resting state - 120 seconds
Frequency-resolved GED components
Explained variance (%)
Frequency (Hz)
Component #1
Component #2
Component #3
Passive listening - 120 seconds
Frequency-resolved GED components
Explained variance (%)
Frequency (Hz)
Component #1
Component #2
Component #3

d

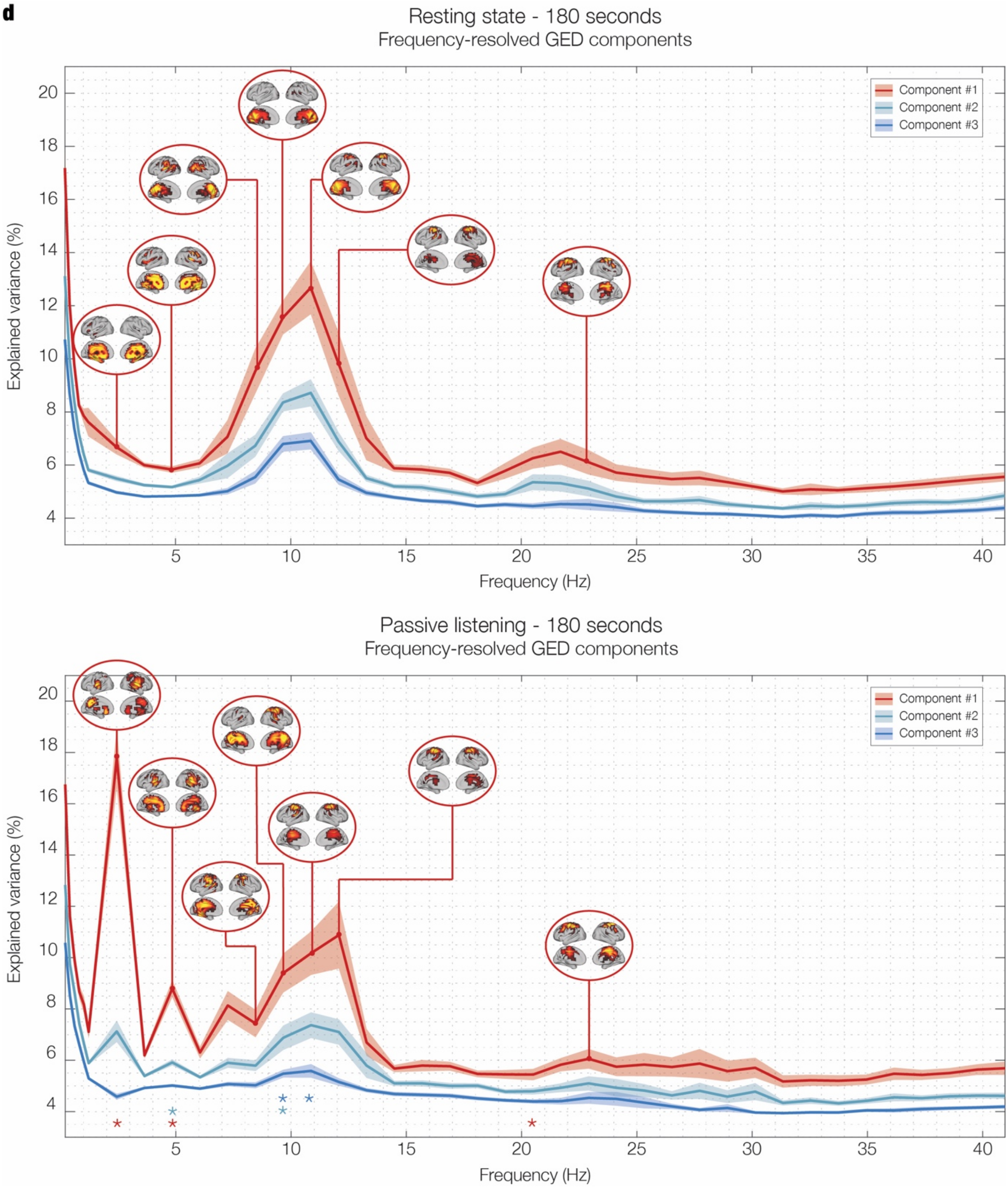
Resting state - 180 seconds
Frequency-resolved GED components
Explained variance (%)
Frequency (Hz)
Component #1
Component #2
Component #3
4
6
8
10
12
14
16
18
20
5
10
15
20
25
30
35
40
Passive listening - 180 seconds
Frequency-resolved GED components
Explained variance (%)
Frequency (Hz)
Component #1
Component #2
Component #3
4
6
8
10
12
14
16
18
20
5
10
15
20
25
30
35
40

e

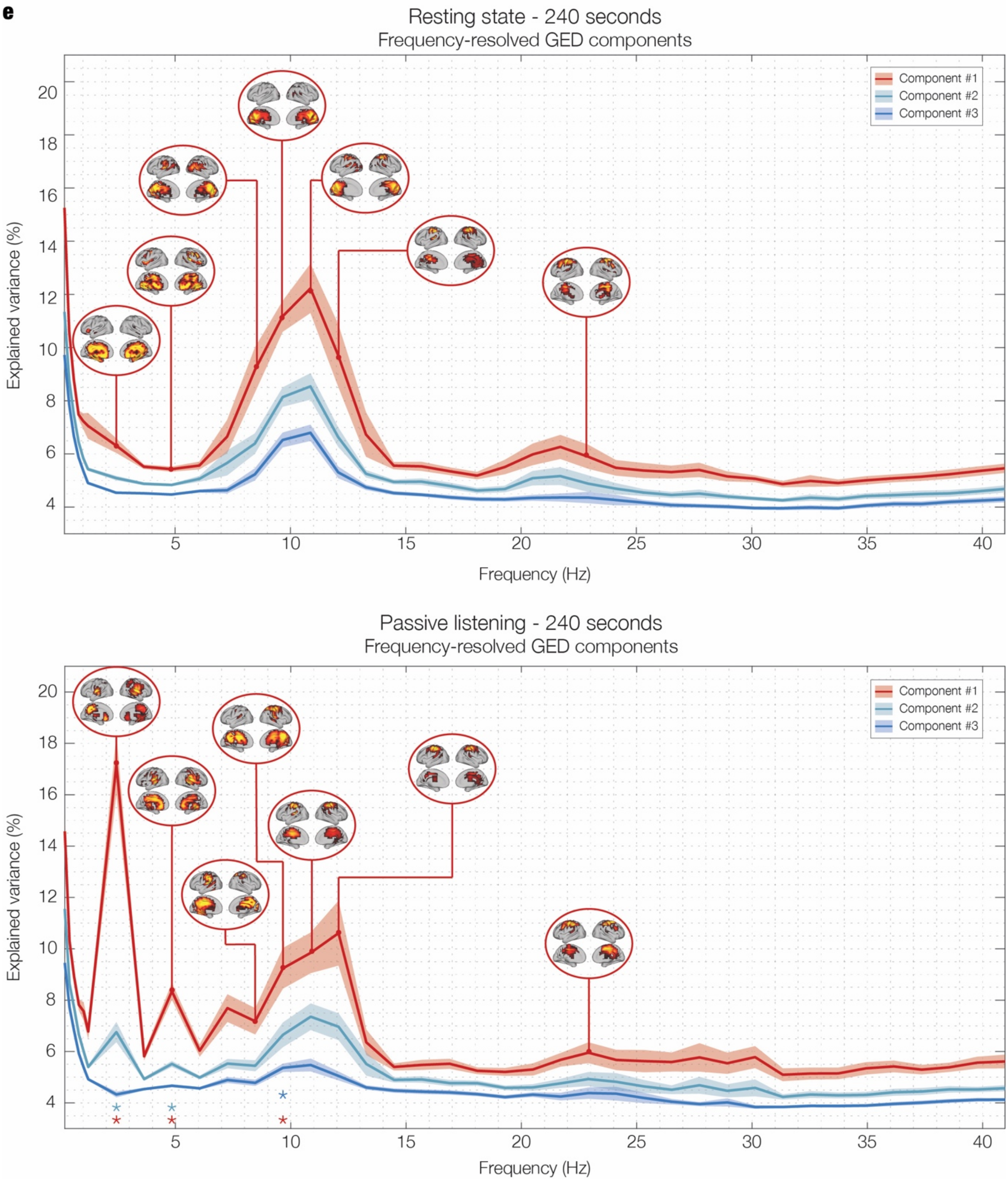

Figure S3a-e. Network landscape: Replication on shorter fragments of the original dataset (RS and PL conditions). *This figure illustrates the frequency-resolved brain networks estimated via FREQ-NESS for the first (a) 30-seconds, (b) 60-seconds, (c) 120-seconds, (d) 180 seconds, and (e) 240-seconds fragments of the RS and PL conditions of our main dataset. The solid lines indicate the mean normalized eigenvalues across participants, while the shaded areas represent the standard error of the mean (SEM). Overall, the main frequency-specific networks and their respective spatial activation patterns were largely replicated, even with dataset fragments as short as 30 seconds. However, it is important to note that for very short fragments (30 and 60 seconds), the overall eigenvalue distribution was affected by the steep 1/f component at the lowest frequencies. This effect only concerned the first two lowest frequencies and is likely an artifact due to insufficient signal duration to reliably estimate these low frequencies.* ***a - 30 seconds****: In RS and PL conditions,*

significant changes were observed in the first eigenvalue at the stimulation frequency (2.4 Hz; $p < 0.001$, FDR-corrected) and its first harmonic (4.8 Hz; $p < 0.001$, FDR-corrected), indicating the emergence of auditory networks even with minimal data input. Here, the two lowest frequencies exhibited a steep 1/f decay. ***b - 60 seconds****: The results mirrored those for the 30-second segment, with significant changes in the first eigenvalue for 2.4 Hz and 4.8 Hz. However, these effects remained constrained to the auditory network. At this duration, only the lowest frequency exhibited a steep 1/f decay, supporting the notion that this feature is most likely an artifact of the input's limited duration.* ***c - 120 seconds****: Significant changes extended to the first three eigenvalues across multiple frequencies, including 2.4 Hz, 4.8 Hz, 7.2 Hz, 9.6 Hz, and 10.9 Hz. With this duration, more nuanced network dynamics started to converge with the original results, including the suppression of alpha networks.* ***d - 180 seconds****: Significant changes were identified in the first three eigenvalues, replicating the patterns seen at 120 seconds and additionally capturing effects at 20.5 Hz. The results demonstrated greater consistency with the full-length recordings.* ***e - 240 seconds****: A near-exact replication of the full-length results was achieved, including significant changes in the auditory, alpha, and beta networks. This duration provided the most comprehensive representation of network landscapes. In conclusion, the main features of the brain landscape within conditions and the reorganization of the landscape across conditions converged at 120 seconds. As a general guideline to implement the FREQ-NESS pipeline in the context of a larger experimental design, we recommend 120 seconds as a minimum duration and 240 seconds for full convergence of the results.*

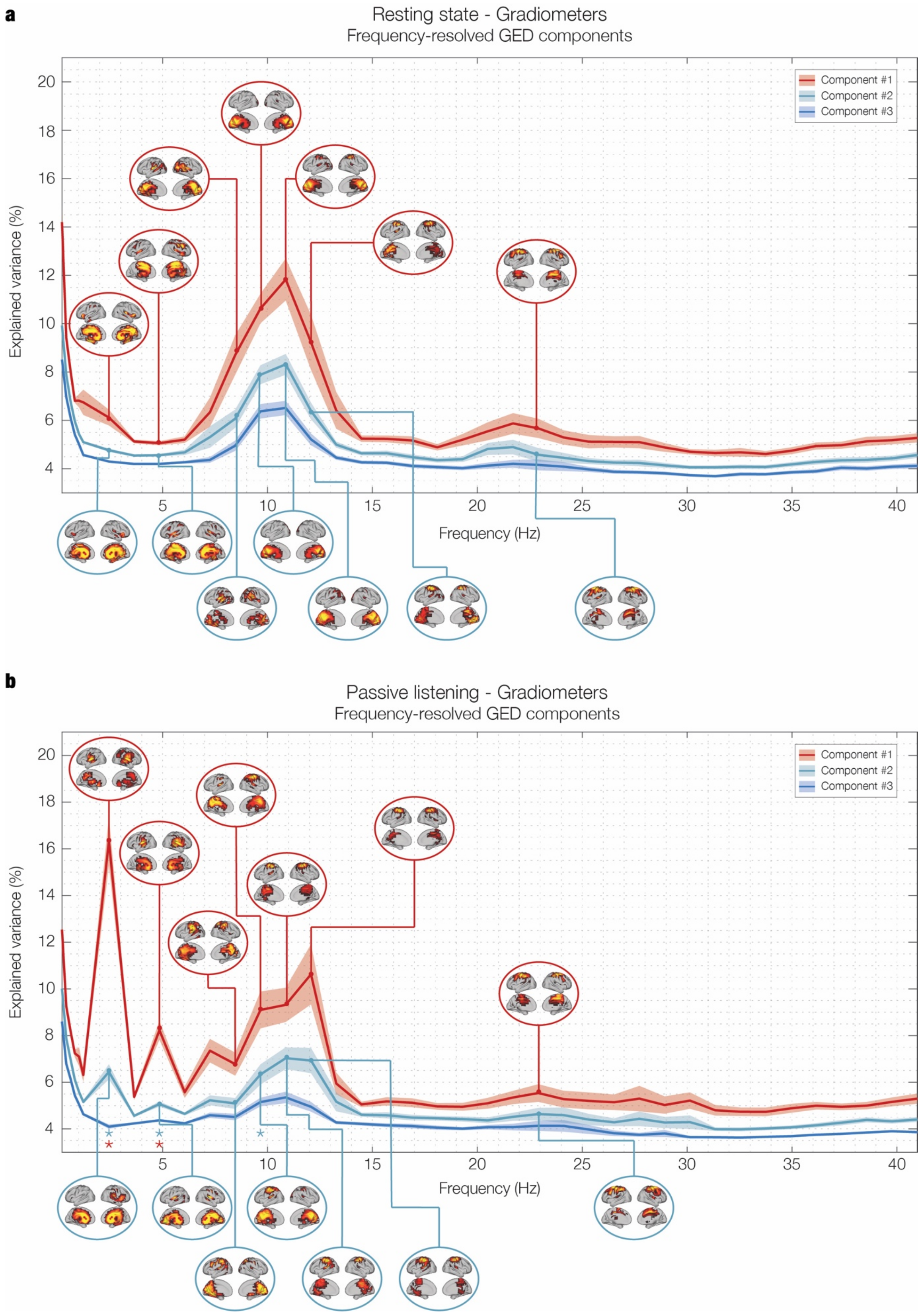
a
Resting state - Gradiometers
Frequency-resolved GED components
Component #1
Component #2
Component #3
Explained variance (%)
20
18
16
14
12
10
8
6
4
5
10
15
20
25
30
35
40
Frequency (Hz)
b
Passive listening - Gradiometers
Frequency-resolved GED components
Component #1
Component #2
Component #3
Explained variance (%)
20
18
16
14
12
10
8
6
4
5
10
15
20
25
30
35
40
Frequency (Hz)

Figure S4. Network landscape: Replication on source reconstructed data from gradiometers (RS and PL conditions). *This figure illustrates the frequency-resolved brain networks estimated via FREQ-NESS for the RS and PL conditions, applying the pipeline to source reconstructed data from gradiometers in our main dataset. The solid lines indicate the mean normalized eigenvalues across participants, while the shaded areas represent the standard error of the mean (SEM).* ***a - Resting State (RS)****: The network landscape reveals a 1/f exponential decline in explained variance within the low delta range and distinct peaks in the alpha (10.9 Hz) and beta (22.9 Hz) frequency ranges. The spatial activation patterns display the characteristic topographies associated with the brain at rest, including the broad mesial distribution linked to the DMN at low frequencies not involved in stimulus processing (2.4 Hz and 4.8 Hz), parieto-occipital activation for the alpha network (10.9 Hz), and sensorimotor engagement for the beta network (22.9 Hz). Interestingly, frequencies just below the alpha peak demonstrate parieto-occipital activation (8.4 Hz and 9.6 Hz), whereas higher frequencies within the range are dominated by sensorimotor activation (12.1 Hz). In the beta range, the explained variance reaches a peak at 22.9 Hz, with the spatial pattern indicating activation confined to sensorimotor areas.* ***b - Passive Listening (PL)****: Significant reorganization of brain networks is observed in response to auditory stimulation. The eigenspectrum reveals a distinct peak at the stimulation frequency of 2.4 Hz and its first harmonic at 4.8 Hz, representing the emergence of networks attuned to the rhythmic auditory input. Spatial activation patterns at 2.4 Hz highlight focused engagement of the right auditory cortex, particularly in Heschl's gyrus, alongside broader medial temporal activation at 4.8 Hz, indicative of secondary auditory processing. Another notable change in this condition is the shift in the alpha peak from 10.9 Hz in the resting state to 12.1 Hz during listening, accompanied by a posterior-to-anterior shift in spatial activation from parieto-occipital to sensorimotor regions. This reorganization likely reflects increased sensory readiness and action preparation in response to the rhythmic stimulation. The beta band remains consistent across conditions, both in terms of eigenspectrum and spatial distribution. Taken together, the replication of the two conditions confirms that the outputs of the FREQ-NESS pipeline are invariant to the choice of MEG sensors to perform anatomical source reconstruction via beamforming.*

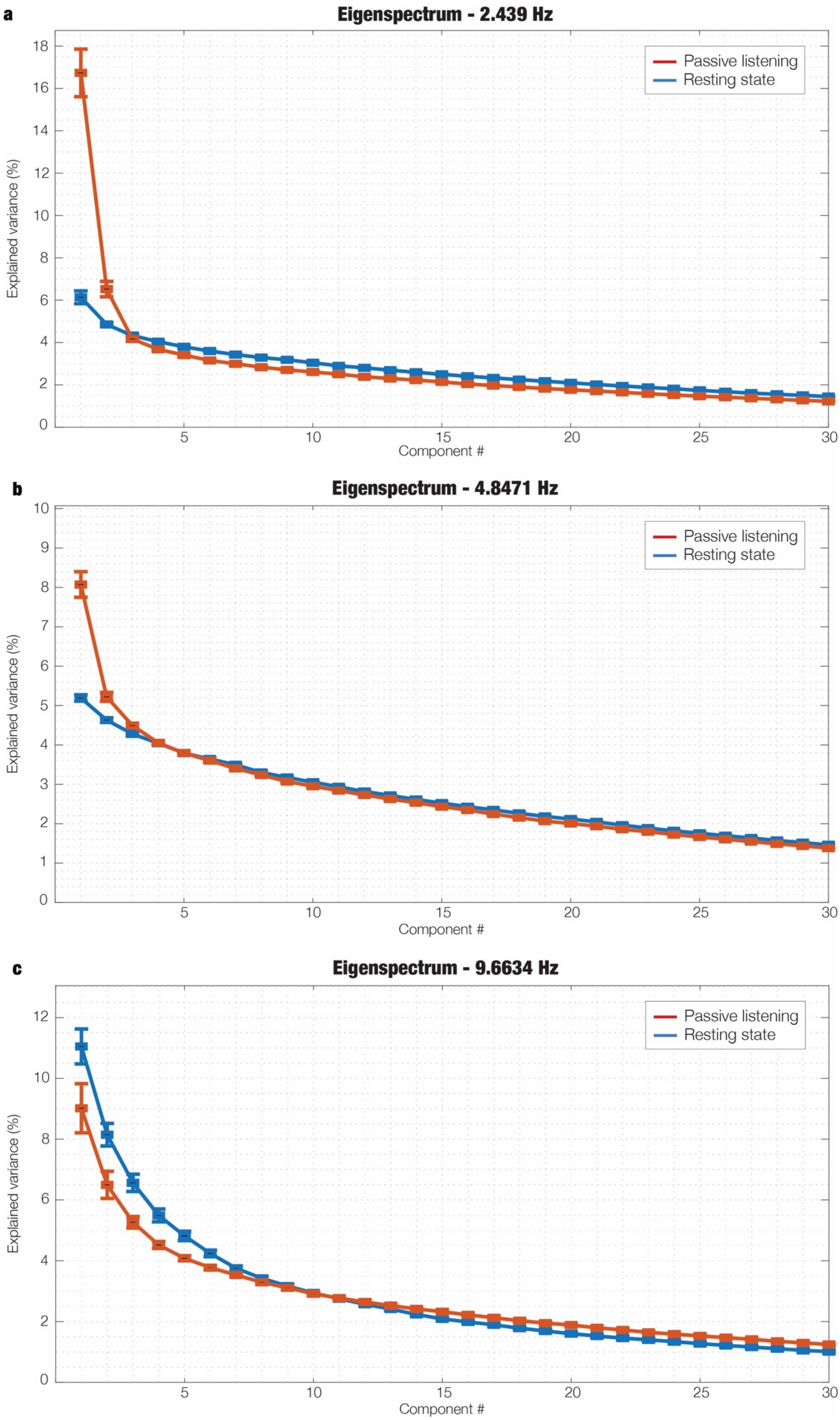
a
Eigenspectrum - 2.439 Hz
Passive listening
Resting state
Explained variance (%)
Component #
b
Eigenspectrum - 4.8471 Hz
Passive listening
Resting state
Explained variance (%)
Component #
c
Eigenspectrum - 9.6634 Hz
Passive listening
Resting state
Explained variance (%)
Component #

Figure S5. Eigenspectra: Eigenvalue distribution for selected frequencies

This figure illustrates the distribution of the top 30 eigenvalues for selected frequencies, normalized to express the percentage of variance explained by each component and sorted in descending order. The displayed frequencies were chosen for their relevance in relation to the aims of the study. Within each plot, eigenspectra are shown for both RS and PL conditions (in blue and red, respectively). ***a -*** *Stimulation frequency (2.4 Hz);* **b** *– First harmonic of the stimulation frequency (4.8 Hz);* **c** *– Alpha peak frequency modulated by the auditory stimulation (9.6 Hz). The characteristic exponential decay of the eigenspectra, which indicates successful source separation, shows that the top components account for a disproportionately high amount of variance. As the component index increases, the variance explained by the components converges exponentially toward zero. This justifies focusing on the leading eigenvalues in our analysis, thereby reducing data dimensionality while ensuring methodological parsimony. Error bars represent the standard error of the mean (SEM).*

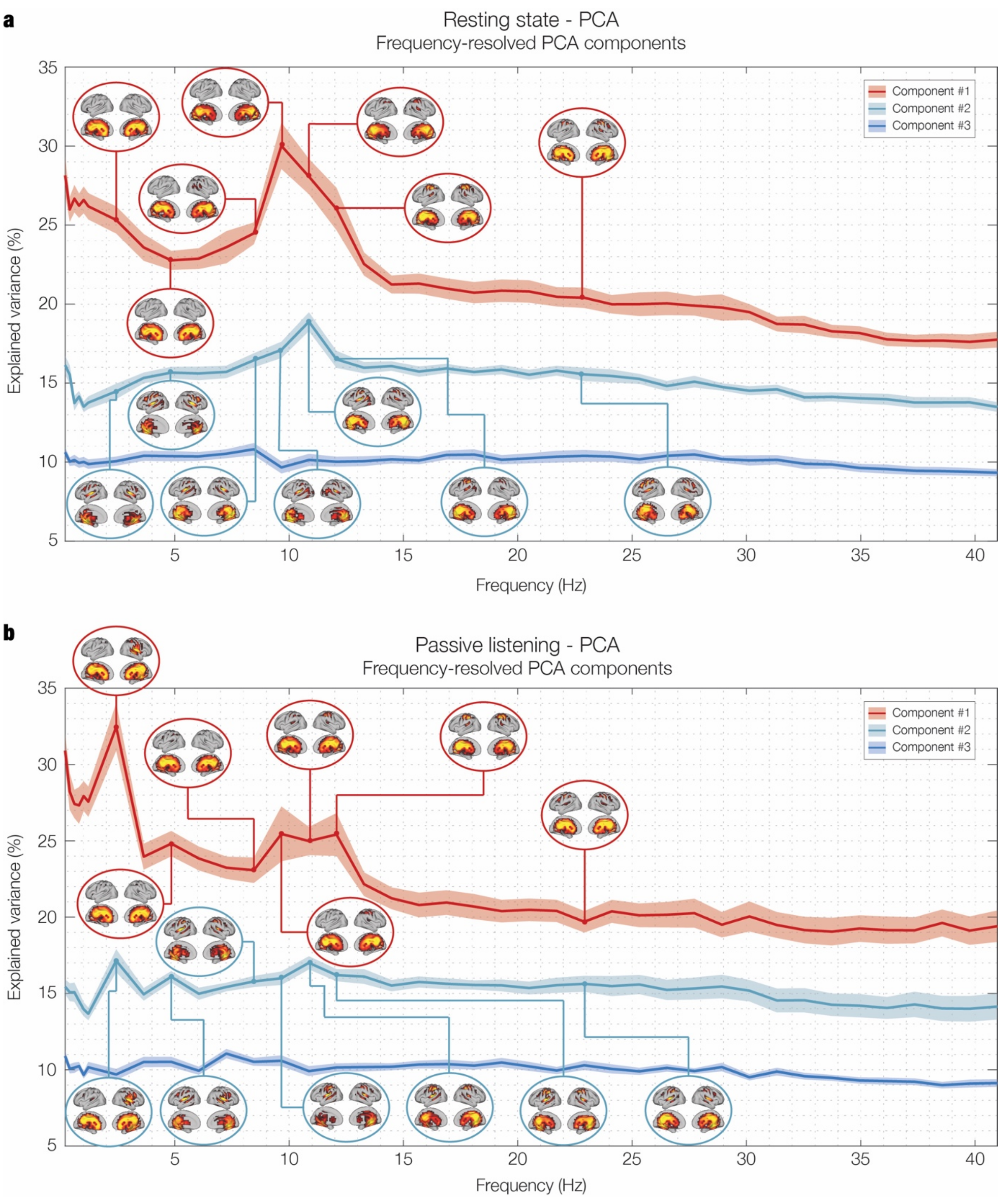

Figure S6. Network landscape: Alternative FREQ-NESS pipeline using PCA (RS and PL conditions).

This figure illustrates the frequency-resolved brain networks estimated via FREQ-NESS for the RS and PL conditions, applying PCA to the source-reconstructed main dataset after narrowband filtering for each frequency bin. The solid lines indicate the mean normalized eigenvalues across participants, while the shaded areas represent the standard error of the mean (SEM). ***a - Resting State (RS)****: The network landscape exhibits a sustained 1/f component in explained variance across all frequencies. Local maxima are still observed in the alpha (9.6 Hz) and beta (22.9 Hz) frequency ranges, though they are less pronounced compared to the GED-derived landscape. This reduced prominence highlights a key limitation of PCA, which operates solely on narrowband covariance matrices without contrasting narrowband data against*

broadband data. As a result, PCA maximizes variance within narrowband data rather than optimizing for frequency-specificity. Additionally, while GED produces smooth, Gaussian-like eigenvalue distributions in the alpha range, PCA instead yields skewed curves, suggesting reduced precision. Moreover, the spatial activation patterns at each frequency exhibit heavily mixed topographies, where the typical DMN configuration persists on the background of frequency-specific activity, thereby hindering its spatial disentangling. ***b - Passive Listening (PL)****: While the reorganization of brain networks in response to auditory stimulation remains, it is distorted by the sustained 1/f component across all frequencies. Similar to GED, the PCA eigenspectrum displays a peak at the stimulation frequency of 2.4 Hz, reflecting the emergence of auditory networks. However, the secondary auditory network, which is distinctly isolated at 4.8 Hz in GED, is barely detectable using PCA. Given that the stimulation frequency and its harmonic should be the most dominant effects in PL, their weaker visibility suggests that PCA's ability to isolate these networks is compromised by the presence of an aperiodic component. Notably, whereas GED sorts components unmistakably based on frequency specificity, PCA is less reliable in correctly attributing networks to the expected eigenvalue index. Concerning the spatial activation patterns, the activation of the Heschl's gyrus is the only focus of activation clearly comparable with the topographies produced by GED, although still mixed with the background DMN configuration. Given that PCA struggles to separate even the most prominent effect in our experimental design—i.e., the emergence of auditory networks—its suitability for frequency-resolved blind source separation in FREQ-NESS is questionable. Real frequency-specific dynamics may be misleadingly assigned to lower-ranking eigenvalues, reducing interpretability. This problem worsens for more subtle dynamics, such as the reconfiguration of alpha networks, where different portions of the frequency range are split between the first and second eigenvalues, disrupting the clear structure observed with GED (see* ***Figure 2****). No significant differences were found across conditions when applying an FDR-corrected one-tailed signed-rank Wilcoxon test (N = 26). However, the observed trends and effect directions align with those produced by GED. Based on these observations and the corresponding quantitative analyses, we conclude that PCA yields a suboptimal solution for isolating frequency-specific networks. In contrast, GED provides superior separation with greater clarity and accuracy.*

References

1. Bonetti, L. *et al.* Spatiotemporal brain hierarchies of auditory memory recognition and predictive coding. *Nat. Commun.* **15**, 4313 (2024).
2. Bruzzone, S. E. P. *et al.* Dissociated brain functional connectivity of fast versus slow frequencies underlying individual differences in fluid intelligence: a DTI and MEG study. *Sci. Rep.* **12**, 4746 (2022).
3. Cohen, M. X. Analyzing neural time series data: theory and practice. (2014).
4. Cohen, M. X. Comparison of linear spatial filters for identifying oscillatory activity in multichannel data. *J. Neurosci. Methods* **278**, 1–12 (2017).
